# Strengthening population health interventions: developing the CollaboraKTion Framework for Community-Based Knowledge Translation

**DOI:** 10.1186/s12961-016-0138-8

**Published:** 2016-08-30

**Authors:** Emily K. Jenkins, Anita Kothari, Vicky Bungay, Joy L. Johnson, John L. Oliffe

**Affiliations:** 1 University of British Columbia, School of Nursing, T201-2211 Wesbrook Mall, Vancouver, British Columbia V6T 2B5 Canada; 2 Western University, School of Health Studies, Arthur and Sonia Labatt Health Sciences Building, Rm. 222, London, Ontario N6A 5B9 Canada; 3 Simon Fraser University, Office of the Vice-President, Research, 8888 University Drive, Burnaby, British Columbia V5A 1S6 Canada

**Keywords:** Knowledge translation, Community-based knowledge translation, Integrated knowledge translation, Population health, Public health, Community, Framework

## Abstract

**Background:**

Much of the research and theorising in the knowledge translation (KT) field has focused on clinical settings, providing little guidance to those working in community settings. In this study, we build on previous research in community-based KT by detailing the theory driven and empirically-informed CollaboraKTion framework.

**Methods:**

A case study design and ethnographic methods were utilised to gain an in-depth understanding of the processes for conducting a community-based KT study as a means to distilling the CollaboraKTion framework. Drawing on extensive field notes describing fieldwork observations and interactions as well as evidence from the participatory research and KT literature, we detail the processes and steps undertaken in this community-based KT study as well as their rationale and the challenges encountered. In an effort to build upon existing knowledge, Kitson and colleagues’ co-KT framework, which provides guidance for conducting KT aimed at addressing population-level health, was applied as a coding structure to inform the current analysis. This approach was selected because it (1) supported the application of an existing community-based KT framework to empirical data and (2) provided an opportunity to contribute to the theory and practice gaps in the community-based KT literature through an inductively derived empirical example.

**Results:**

Analysis revealed that community-based KT is an iterative process that can be viewed as comprising five overarching processes: (1) contacting and connecting; (2) deepening understandings; (3) adapting and applying the knowledge base; (4) supporting and evaluating continued action; and (5) transitioning and embedding as well as several key elements within each of these processes (e.g. building on existing knowledge, establishing partnerships). These empirically informed theory advancements in KT and participatory research traditions are summarised in the CollaboraKTion framework. We suggest that community-based KT researchers place less emphasis on enhancing uptake of specific interventions and focus on collaboratively identifying and creating changes to the contextual factors that influence health outcomes.

**Conclusions:**

The CollaboraKTion framework can be used to guide the development, implementation and evaluation of contextually relevant, evidence-informed initiatives aimed at improving population health, amid providing a foundation to leverage future research and practice in this emergent KT area.

## Background

Knowledge translation (KT) has become an increasingly popular area of focus within the health community, garnering significant attention and support from scholars, policymakers and research funding agencies. Despite enthusiasm surrounding the science and practice of KT, critiques have emerged. One notable concern pertains to the narrow focus of KT research and theorising, which has predominantly centred on enhancing the use of scientific evidence among health practitioners working in clinical settings. Little attention has been given to the study of KT approaches aimed at improving health outcomes beyond acute and primary care environments [1]. Recognising this gap, scholars [1–3] have advocated for a broadened scope within KT science and practice – one that focuses on improving health outcomes within community settings and/or among populations. Such approaches are informed by a public health perspective incorporating health promotion and disease prevention. This emergent area of KT science and practice, often referred to as community-based knowledge translation (CBKT), requires further development [4]. The purpose of this article is to advance CBKT by describing the processes underpinning a CBKT study as a means to guiding future work in the area.

### Community-based knowledge translation: an emergent area of KT science and practice

Over the last decade, the knowledge and science regarding CBKT has been steadily evolving. In 2010, Wilson et al. [3] proposed a framework for conducting CBKT, recognising that community-based organisations represent an important component of the healthcare system. Drawing on their definition of community-based organisations as non-governmental organisations, grassroots societies, and civil society groups, Wilson and colleagues’ framework included four components: (1) the development of partnerships; (2) the production of systematic reviews addressing community relevant issues; (3) the creation of an online information portal to house evidence from systematic reviews; and (4) utilisation of ‘rigorous’ evaluation approaches, including RCTs augmented by qualitative data to explore the impact of CBKT efforts [3]. While these authors were among the first to identify the need for CBKT approaches in order to more comprehensively address the health of populations, their work provided limited direction for applying the framework.

Kothari and Armstrong [1] pushed the conceptualisation of CBKT further by identifying the importance of intersectoral collaboration and highlighting the need to focus on health promotion and disease prevention. They suggest that, although diverse, community-based settings shared the following characteristics, rendering KT needs in these environments unique:

- They typically target networks of multiple organisations and stakeholder groups across sectors who work in collaboration (whereas clinically focused interventions are usually aimed at individuals or single organisations);
- The types of knowledge valued by the diverse groups of stakeholders involved in community settings (e.g. members of public health departments, non-governmental organisations, health authorities) are broad and include experiential and local knowledge (whereas, in clinical settings, the types of knowledge utilised by KT researchers tends to be narrow and focused); and
- Advocacy serves as a central KT activity in the community, a role that has not been proposed by traditional KT scholars.

Additionally, CBKT holds the potential to address issues using a population or public health approach, as opposed to the curative approach typical of clinical settings.

**Fig. 1:**
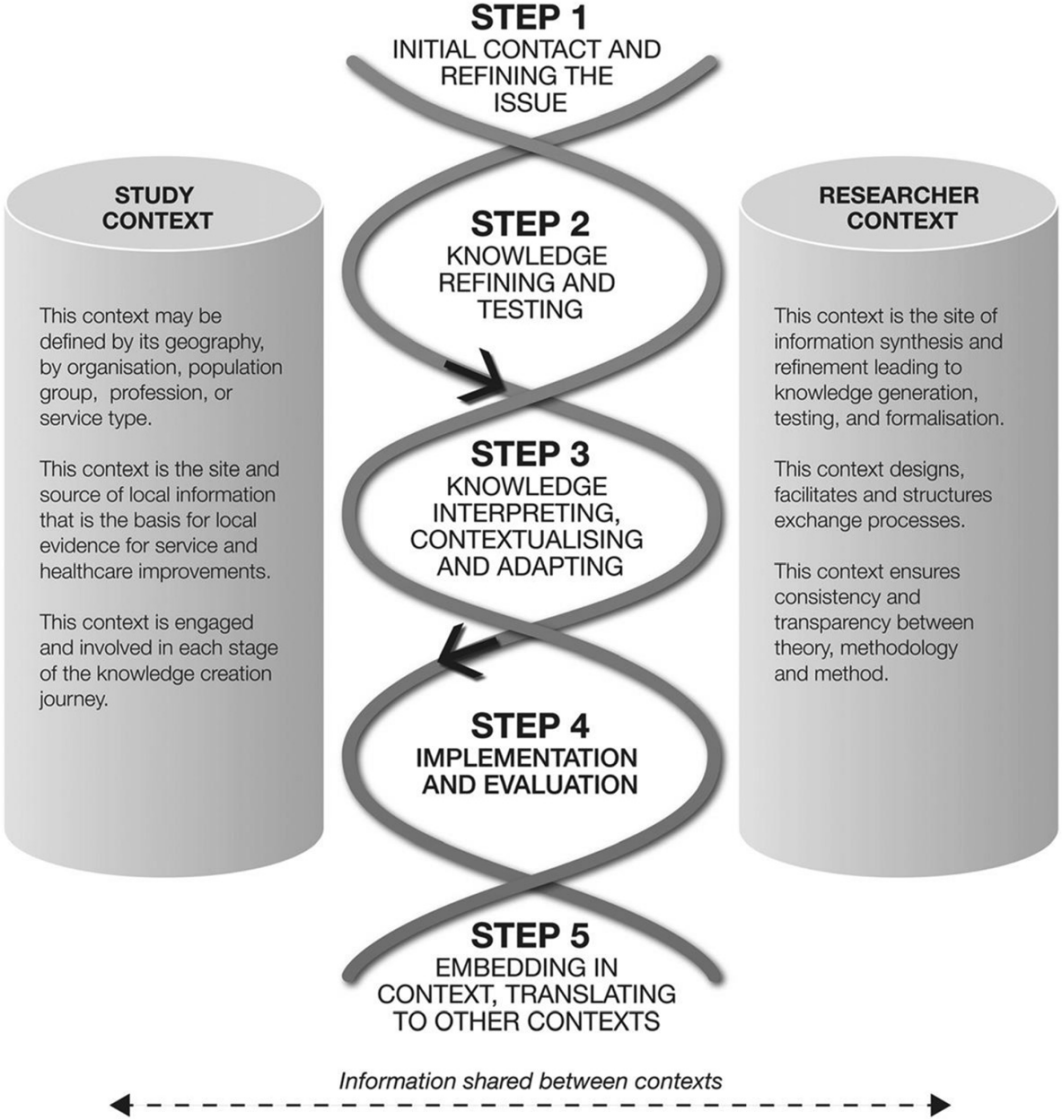
Co-KT Framework for conducting population focused KT. Produced by Kitson et al. [2]

Although the work of Wilson et al. [3] and Kothari and Armstrong [1] initiated dialogue regarding the need for expanded KT goals, intervention targets and approaches, there remained a lack of guidance for conducting KT initiatives in community-based settings. Responding to this need, scholars have put forth conceptual frameworks to further inform the CBTK process (e.g. [2, 5]). Campbell [5], for example, drew on the theoretical underpinnings of participatory research and the Ottawa Model of Research Use to develop a conceptual framework for CBKT. Campbell’s model, however, lacked the detail required to integrate the approach to inform future work. For example, descriptions of the different components of the framework or empirical examples of how the various stages of the framework are enacted in context are needed to facilitate application of this approach. More recently, Kitson et al. [2] presented the ‘co-KT’ framework, a guide to conducting KT within a population health study. Co-KT was presented as a collaborative approach informed by the theoretical foundations of engaged and participatory scholarship, and academic–community partnerships. The authors describe co-KT as “*a framework for actioning the intent of researchers and communities to co-create, refine, implement and evaluate the impact of new knowledge that is sensitive to the context (values, norms, and tacit knowledge) where it is generated and used*” [2] (p. 3). The framework proposed five steps embedded within an overarching context. This context was described as including the ‘study context’ (i.e. the study site or location, stakeholders, local information and expertise) and the ‘research context’ (i.e. researchers who facilitate the study and ensure scientific integrity throughout the co-KT process) (Fig. 1). Subsequent exploration of this framework by the authors in a rural context demonstrated that the co-KT approach was effective for linking local and academic knowledge, but noted that “*full implementation of the co-KT framework was not possible*” [6] (p. 1).

The co-KT framework [2] provided important direction for engaging in population-focused KT. In addition, the authors raised an issue of critical importance to the practice of CBKT – the nature of knowledge valued and utilised. Given the heterogeneous nature of community-based settings, Kitson et al. [2] also acknowledged that creating change to improve health within communities and/or among populations necessitated the incorporation of a diverse knowledge base, one that drew on a more universal knowledge that could be transferred between settings with little need for adaptation, as well as knowledge that built on the various needs, expertise and contexts of local stakeholders.

The contributions made by these and other scholars in the emerging area of CBKT have provided a foundation upon which to build. From this literature, the unique nature of CBKT became evident – the population and/or public health focus and related practices of health promotion and disease prevention distinguishing CBKT within the broader KT field were also illuminated to highlight the need to develop specific KT methods for community settings. Furthermore, the collaborative approaches requisite to community-based work indicated that CBKT was a form of integrated KT – a process underpinned by tenets of participatory research [7]. Indeed, scholars have pointed to participatory research as a key paradigm informing this approach (e.g. [8, 9]).

While existing CBKT frameworks provided important guidance to scholars working in the field, there remains a paucity of literature detailing their intricacies and potential applications. There is also a need for empirically based methodological guidance to determine and formally evaluate the expected outcomes of CBKT as a means to refining existing frameworks. In this article, we address these gaps by describing the processes in conducting, and the empirical products derived from, an ethnographic CBKT study focused on youth mental health promotion.

## Methods

A case study design was utilised to uncover the processes involved in conducting a CBKT initiative. This design was selected because it promotes rich comprehension of complex, socially situated phenomena or practices [10]. Ethnographic approaches facilitated a detailed understanding of CBKT processes in context [11]. The site for this study was a community we refer to by the pseudonym, Lakeview. Lakeview is a rural, resource-oriented town in north-central British Columbia, Canada. This study site was selected based on previous qualitative research that our team had conducted in the community exploring the ways in which context influences young peoples’ experiences of emotional distress. This initial study afforded opportunities to build collaborative research relationships and gain an understanding of the profound need for community-driven initiatives targeting youth mental health in this community [12]. This research also provided a rich source of local knowledge to underpin the CBKT initiative.

From April 2013 to September 2014, our research team worked with the Lakeview community on the *Social Networking App/Action for Resilience* (SONAR) initiative. Study collaborators initially consisted of 10 youths (aged 13–18 years) who attended the local high school. These young people applied to become ‘youth collaborators’ (YCs) for the study and were selected to ensure diversity in age, gender, and ethnicity to promote the reach of the initiative developed. Given the health promotion focus of CBKT work, the YCs had a range of experiences with mental health challenges; however, personal experience was not a prerequisite for involvement. Adult stakeholders included school administration and leadership, teachers and counsellors, District of Lakeview staff, members of city council, representatives from one of the local First Nations, and community youth workers. During the study period, the research team made six visits to the community, each ranging from 3 to 6 days in duration. In addition, the research team and YCs met via videoconference on a weekly basis to move the project forward.

### Data sources and analysis

At the outset of the study, we established a guiding framework which included anticipated outcomes based on findings drawn from our examination of systematic review data from the participatory research literature in the area of population health. Potential outcomes to be achieved through a CBKT process included enhanced leadership and community capacity, civic engagement and advocacy, shifting community practices and norms, health behaviour change, empowerment, gains in self-esteem, positive self-concept, and enhanced knowledge [13–15].

After reviewing the relevant literature and identifying a study plan, we began the CBKT processes. Data for this component of the study were derived from extensive field notes and email correspondence compiled by the lead author (EKJ). These field notes consisted of 65 entries spanning 88 pages and documented fieldwork observations and interactions, detailing the steps undertaken during the project and their rationale, and describing the challenges encountered. Field notes were documented using word processing software to facilitate analysis. While our project was underway, the co-KT framework proposed by Kitson et al. [2] was published. In keeping with the iterative and responsive nature of community-based research and our desire to situate the current work within the evolving field of CBKT, we decided to be guided by the five steps of the co-KT framework in analysing the data we had collected. This approach supported us in applying an existing CBKT framework to the data and provided an opportunity to contribute to the literature on the theory and application of CBKT through an inductively derived empirical example. Field notes were read several times and coded according to the co-KT steps. Each code was then organised to provide a detailed description of the processes undertaken in each stage of this CBKT process. Sub-codes were later established to highlight key elements of the process that occurred within each of the five steps. EKJ completed the initial coding procedure and author agreement was reached through an iterative process of discussion and returning to the data.

Ethical approval for this project was obtained from the University of British Columbia, Behavioral Research Ethics Board (H13-00733) and all participants provided written consent prior to participation in the study.

## Results

Drawing on the five steps of the co-KT framework (Fig. 1), the findings describe the processes undertaken throughout the initiative, highlighting the strategies used and challenges experienced. In addition, sub-headings have been used to highlight new, empirically derived elements of the CBKT process identified through our analysis. While these data are presented in a step-by-step fashion for the purposes of clarity, the processes were iterative and non-linear.

### Step 1 – Initial contact and refining the issue

Initial contact and refining the issue involved establishing relationships with community partners and determining the nature of the health goal to be addressed. This process served as the foundation upon which the possibility of achieving desired outcomes were built. Kitson et al. [2] described this step as involving data being “*conveyed from the study context to the researcher context in response to a query. The initial query may be generated by either context, but will be formally framed by the researcher context*” (p. 4).

#### Building on local knowledge

As previously noted, we had developed relationships with the community of Lakeview through an earlier project that focused on understanding how context influenced young peoples’ everyday experiences of emotional distress. Through this work we gained meaningful insights into the challenges faced by youth in Lakeview and also established partnerships with community members who wanted to work to improve adolescent mental health. This earlier research culminated in a community report outlining key findings [16]. Given the community’s needs and interests, our query became: how do you collaborate with a community in developing an evidence-informed mental health promotion initiative to improve youth mental health?

When negotiating the process of embarking on the SONAR initiative, we utilised the community report as a strategy to engage stakeholders and demonstrate our desire for a mutually beneficial partnership. This report also served as a valuable source of local knowledge upon which to build a CBKT initiative. Responses to this report were indeed positive. We received emails from youth participants who were pleased to read how their stories had been highlighted and used to draw attention to the issues they were facing. One participant wrote, “*Wow, it’s nice hearing from you. I kind of forgot about this, but* [the report] *was so well done, I love it. Thank you for letting me be a part of it*”. Members of the city council asked to share the report with their stakeholder groups, and school administrators felt that the challenges they faced in addressing youth mental health were validated.

#### Establishing partnerships

We determined at the outset of the project that youth should be the central collaborators. We also decided to make the YC positions a paid role, a gesture to demonstrate the meaningful contribution that we believed young people would make to the project, as well as foster a sense of responsibility for the work that would need to be done. With the assistance of the local high school leadership, advertisements were posted in the school hallways for the position. In May 2013, two members of the study team travelled to Lakeview for the initial SONAR initiative site visit. The goals of this visit were two-fold: to hire YCs and to engage members of the broader community through a forum on youth mental health.

#### Balancing researcher and community contexts

During the initial visit, we interviewed 10 young people who had emailed applications for the YC positions prior to our arrival. To support diversity, we asked school leaders for assistance in recruiting additional applicants (9 of the 10 emailed applications were from girls). Ultimately, we interviewed 25 young people and hired 10 YCs. One of the reasons for the large number of interviews was that school counsellors emphasised the benefits that were derived by young people going through the interview process. These additional interviews served as an opportunity for our team to provide a meaningful service to the community, while also ensuring a group of YCs with a range of interests and experiences. Because of the number of interviews conducted, the hiring process took longer than anticipated, causing one of our team members to document, “*I am a little worried that this is going to become a theme in this work – things taking longer than expected*”, a sentiment that proved true as the study team became further engaged within the community.

Given that the study site was located in a rural town 10 hours’ drive from the research base, much of the project took place virtually. Each YC was given an iPad to use for weekly videoconference meetings and study activities. The YCs were excited about the project, seeing it as an opportunity to gain new skills and make a difference in their community. Following hiring decisions, the study team and YCs met for three in-person afternoon meetings. During these first sessions, the group worked on establishing an identity, team building and visioning, and discussing the types of activities that they might be involved in throughout the project. Some youth asked if they would get to interview their peers like they had seen the research team do when we were conducting our initial study in the community. At this point, one of the researchers noted that the team would have to work on establishing realistic expectations for what could be accomplished while ensuring that the researcher and study goals were met.

#### Challenges in building group cohesion

While the YCs all expressed excitement and motivation to create change to improve mental health in their community, it took time for the group to function cohesively. For example, two of the girls in the group had a history of conflict, which became increasingly evident as the group began working together. At one point, a member of the research team had to discuss the issue with each of the girls and encourage them to keep their personal differences outside of the work setting. One of the other primary challenges that we faced as the study progressed was attendance at weekly videoconference meetings. While the YC position was presented as a formal job and the youth were compensated for their time, attendance at the meetings fluctuated throughout the project from two to 10 YCs per session (mean = 7). Discussions about this issue occurred repeatedly throughout the study period. The research team spoke to the YCs about accountability in the context of employment and explored options to shift the timing of the meetings. Each week, the YCs that were present at the meeting were encouraged to share key updates with their peers and to remind each other to attend the following week. A pro-rated payment structure had to be implemented to reflect involvement. These unforeseen challenges with participation caused discomfort and unease among members of the research team who tried to respond constructively to the emergent dynamics in the community setting. For example, the research team member who facilitated the weekly meetings frequently documented concerns about whether she was “*doing something wrong or not engaging the youth as they wanted to be involved*”. Ultimately, the research team recognised the importance of flexibility when working with young people in this community and identified that one of the key roles of the facilitator would be to foster productivity among the YCs who were in attendance at any given meeting, thus recognising and responding to context.

With ongoing reflection and returning to the data from our initial study, it became clear that the context within which these youth were living impacted their ability to commit to the project in the ways that the research team had expected [12]. Young people growing up in Lakeview faced some challenging circumstances related to issues such as parental substance use, poverty, absence of an adult guardian, and responsibilities for providing income for their family’s food and housing costs, among others. Factors in the study context influence engagement when working collaboratively in community settings, which contributed to our belief in the importance of building strong relationships during the initial contact stage of a CBKT process.

In addition to hiring and beginning to build relationships with YCs, our research team hosted a forum on youth mental health in an effort to share findings from our initial study and to engage potential adult stakeholders in the next stages of the work. This effort was also met with challenges. While we had corresponded with members of city council and school leadership to assist in advertising this event, the message did not go out as discussed. When we expressed concern about limited public awareness of the event, we were told, “*sometimes short notice is better in this town*”. The result of this communication breakdown was limited turnout to the forum (11 community members); however, it remained an important avenue for communicating information about the project and making contact with key stakeholders who would prove to be instrumental in the study process as it continued. Again, ongoing reflection revealed that the context of this town was contributing to the ways in which people engaged with the study. Repeatedly, we were told that the project was of great importance to the community, though commitment was often lacking and communication slow. We identified that the people in Lakeview who had the motivation and resources to contribute to making a better community were overworked and fatigued. One of the high school teachers expressed, “*I would love to be involved, but my plate is already full*”. Other community members expressed similar sentiments. This reality resulted in less support from adult stakeholders than was initially promised or expected.

The stage of initial contact and refining the issue is critical to the success of a CBKT project. Reflecting on our data, we propose that this stage does not occur in isolation from the other stages, but rather represents elements that continue to be revisited throughout the study process. For example, as new stakeholders become involved, the process of initial contact was revisited as the relationship was established. Furthermore, given the iterative nature of community work, there was some degree of adaptation or refining of the study focus occurring throughout the project period.

### Step 2 – Knowledge refining and testing

After re-engaging the study community and strengthening partnerships with key stakeholders interested in research collaboration to enhance youth mental health, we undertook activities associated with the second stage of the co-KT framework – knowledge refining and testing. It is pertinent to note that we had begun steps of knowledge refining and testing as part of our initial contact process, though this action intensified as our study progressed. Kitson et al. [2] described this step as the research team using their skill set to translate data and locally derived evidence into an accessible product by “*considering existing evidence, the perspectives of multiple stakeholders, and the ongoing input from the study context*” (p. 4). While we began this process when initially reconnecting with the community, it continued for a period of approximately 5 months.

#### Gathering and reviewing diverse sources of knowledge and building capacity

During a 5-month period, the research team and YCs met via videoconference for 45–90 minutes on a weekly basis. Given the youth mental health promotion focus of this project, each meeting began with a relaxation exercise or stress management practice (e.g. mindfulness practice, breathing exercise, review of an online mental health resource). The research team initially led these exercises; however, as the YCs became increasingly engaged, they took turns identifying useful practices and leading these session introductions. The meetings then focused on reviewing and reflecting on a variety of sources of knowledge. These included data from the community report, results of an asset mapping exercise undertaken by the YCs identifying sources of strength for youth in the community, scientific evidence pertaining to mental health (e.g. evidence regarding the determinants of mental health and illness, mental health promotion strategies, effective approaches for improving mental health in community settings, research regarding mental health and illness among youth) (e.g. [17–20]). The session introductions also included experiential knowledge from the YCs, and information gathered through discussion with adult stakeholders. Both the study team and the YCs assisted in identifying sources of knowledge or evidence for review. Through this process, the YCs gained experience and confidence in locating and reviewing various forms of evidence to inform project actions.

#### Deepening understandings

The research team shared relevant evidence with the YCs for review. In instances where the original evidence document was written at a level that was not accessible to a lay audience, the research team developed summaries of the material in plain language and at a level appropriate for high school students. The research team facilitated discussions based on the YC’s interpretations and reflections during weekly videoconference meetings. The study context was again influential for how this process unfolded. For example, some students who attend high school in Lakeview experience developmental challenges, which in some cases resulted in challenges with reading comprehension. Late in the knowledge refining process, we became aware that one of our YCs had very poor reading skills. At this point, the group was quite cohesive and supportive so the youth worked together to assist this individual in understanding the content that was being reviewed.

Through the process of knowledge refining and testing, the YCs and research team co-identified key factors influencing the mental health of young people in this community. These issues included substance use, bullying and racism. In addition, a concern that was repeatedly raised in our previous research and by YCs and adult stakeholders was an absence of opportunities for youth to be engaged within their community; to feel valued and to form meaningful relationships with adults and peers – otherwise put, a lack of community connectedness. Through continued discussion and review of scientific evidence on adolescent mental health, the SONAR team determined that this issue was of central importance and likely influenced the mental health problems that the youth had identified.

### Step 3 – Interpreting, contextualising and adapting the knowledge base

After engaging in a lengthy process of reviewing and interpreting evidence and identifying local challenges in light of this knowledge, the SONAR team engaged in activities identified in the third step of the co-KT framework. Kitson et al. [2] describe this step as the point at which “*local evidence is refined and tested against the existing evidence to create intervention ‘prototypes’ to be introduced and tested in the study context*” (p. 4). Based on the evidence review process undertaken during step two, community connectedness was identified as a central challenge in the Lakeview context, with detrimental effects for youth mental health. Connectedness refers to a sense of belonging and attachment to others and has been identified by some researchers as the ‘strongest protective factor’ against a number of mental health challenges and a key contributor to the development of resilience [19–22].

During the SONAR initiative, both youth and adult stakeholders repeatedly described a context where there is “*nothing* [for young people] *to do*”, which was used as an explanation for substance use, vandalism, violence, boredom and lack of ambition. It was further explained, “*if you don’t play sports, then it sucks even more, too, ‘cause there’s not a lot of activities going around in this town*”. Beyond an absence of formal activities for youth to engage in, there were frequent reminders in the community that young people were not valued. Signs were posted in some shops indicating only “*two teenagers allowed at a time*”. Young people did not feel that there were safe places for them to hang out, learn skills or connect with mentors in the community.

#### Creating a vision

To address poor community connectedness, the youth engaged in brain-storming exercises and were encouraged to ‘think big’ about the types of initiatives that could be developed to address this concern. This task turned out to be challenging. The YCs had great difficulty coming up with ideas, and when they did, they quickly identified reasons why they would be “*impossible*”. In an attempt to understand this experience, our research team returned to the data. Reflecting on stories shared by youth participants during our initial study, we were able to see how the context in which these young people were growing up was influencing their ability to imagine new solutions. In this community, youth were not routinely exposed to opportunities or engaged in discussion about possibilities for their future. When asked about life goals, many youth struggled to identify their ambitions. In an attempt to address this challenge, the research team distributed a report by Tolman et al. [23] entitled, “Youth Acts, Community Impacts: Stories of Youth Engagement with Real Results”. This document detailed case examples from around the world where the actions of young people had resulted in community transformation, providing a source of inspiration for what could be achieved through the efforts of a group of dedicated youth. After engaging in this process for a period of approximately 3 months, the YCs were able to build on existing evidence as well as their experiential knowledge to identify a focus for their initiative – they decided to develop and disseminate a smartphone/computer ‘app’, henceforth referred to as the ‘app’, which could be used to facilitate engagement in youth-relevant activities available in Lakeview. At this time, the YCs also identified a name for their project, the Social Networking App/Action for Resilience initiative (SONAR).

#### Developing the ‘formal intervention’

The app proposed by the YCs was aimed at fostering community connectedness and consisted of three main components: a ‘real time’ database of opportunities for youth; a place for users to post ideas for positive change in their community, the details of which would be relayed to relevant stakeholders (e.g. city council, local First Nations) to help shape policy and programs impacting youth; and links to online resources aimed at supporting youth mental health. By fostering youth engagement through the use of youth-relevant technology, the YCs believed that community connectedness could be enhanced and that community factors contributing to mental health challenges among young people could be improved. Furthermore, although this project was carried out in a rural community with a high degree of poverty, the majority of youth had access to a smart phone or computer-based internet.

In addition to the functions of the app itself, it provided a platform to spark dialogue about young peoples’ needs in this community and supported the development of additional opportunities for positive engagement. At this point in the process, the SONAR team arranged to share the work done to date with the Lakeview City Council. The intention of this presentation was to begin to publicise the youth collaborators’ idea for an evidence-informed mental health promotion intervention, while also providing the youth with an opportunity to build capacity in the areas of presentation skills and civic engagement. Three of the YCs presented at the council meeting held in September 2013. The idea was so well received that the District of Lakeview offered to provide the funding necessary to hire a web developer to move the project forward and build the app.

**Fig. 2:**
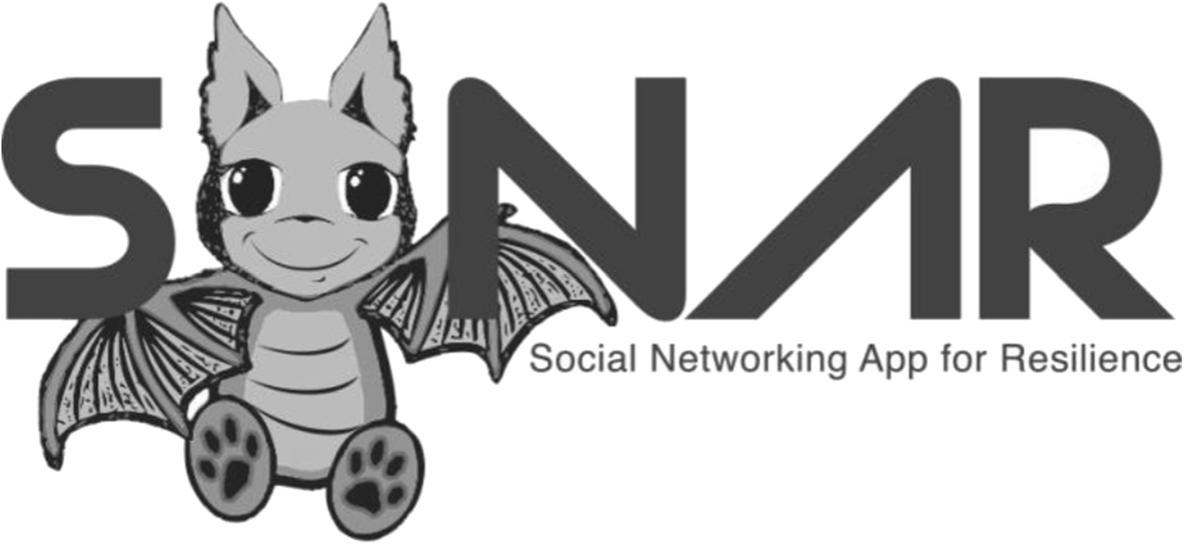
SONAR Project branding created in partnership with Youth Collaborators

Having received funding to move towards implementation, weekly meetings focused on further solidifying a team identity, building the app, branding and identifying a dissemination strategy; a process that took approximately 4 months. A graphic designer was engaged to produce a project logo using the YC’s ideas and drawings (Fig. 2). A web developer was hired to work with the youth to develop the content and aesthetic of the app. In addition, the youth received training from the web developer in managing the backend of the website so that they could continue to add content and make changes to the app as the project progressed. The intention of this training was to empower the YCs with the skills to manage their project and to provide them with experience in website management, which they could use for future work opportunities.

### Step 4 – Implementing and evaluating

Following the inductive design of the formal project initiative, the team worked for the following 4 months on a process of implementation and concurrent evaluation (evaluation data will be presented in a forthcoming article). In the co-KT framework, this step was described as involving the community in implementing the intervention, assessing the impact, and making necessary modifications in order to enhance the intervention and support sustainability. With the web app developed, the YCs and research team focused on dissemination and uptake of the intervention. In an effort to foster intervention uptake and promote sustainability of the project, the team hired five additional YCs. The youth arranged to make a presentation at a school assembly to ‘unveil’ their project. Each of the YCs spoke during this presentation, sharing information about mental health and the mental health challenges they have witnessed in their community and discussing why it is important to address this important public health issue. One collaborator shared her personal story about struggles with mental illness. Against this backdrop, the youth presented their app, demonstrating the components and how to access it.

#### Planning for dissemination and promoting uptake

In the weeks following this presentation, the youth set up booths in the school hallway at lunch time and offered assistance in downloading the app to their peers’ smartphones. Prizes, which consisted of promotional materials that the youth had helped design (wristbands and beanies embroidered with the study logo), were used as an incentive to download the app and to raise awareness about the project. The YCs were also invited by local community groups to set up booths and give presentations about SONAR at two community events: a winter festival and a career fair. The youth were provided with honoraria for participating in these events, which was put into an account to support ongoing action. The local newspaper featured two stories about the project, further promoting the SONAR initiative. Between January and June 2014, activity on the web app was monitored using Google Analytics, which identified 158 unique visitors and 280 sessions, with an average site viewing of approximately 4 minutes. Uptake did not occur as quickly as anticipated. While research indicates that adoption of new interventions can be a slow process that is dependent on a number of characteristics within the study context [14, 24], we were interested in engaging the YCs in discussing their interpretation of this challenge. At this point in the CBKT process, the youth were feeling defeated – their hard work had not resulted in the enthusiasm that they had hoped to see among their peers. While the YCs were struggling to promote uptake of the app among their peers, the research team was challenged by the YCs’ lack of commitment to maintain the app. The real-time database of opportunities for young people, which was a primary feature of the app, required daily updating by the youth who struggled throughout the project period to fulfil this need.

#### Shifting community practices

Despite the challenges, there were signs that positive shifts were occurring within the Lakeview community. For example, young people started to be invited to participate on committees that shape the town’s future and the District of Lakeview began to host committee meetings at the high school to facilitate youth participation. Two YCs joined the local Arts Council to focus on developing arts-based opportunities for young people. With the persistence of the YCs, new youth-focused opportunities were developed, including a yoga class for youth and a First Nations’ traditional arts and crafts program. Stakeholders shared stories with the research team about changing language among young people regarding contentious issues within the community such as sexual orientation. The YCs applied for and secured grant funding to support the creation of a youth-led documentary highlighting positive activities and opportunities for youth in Lakeview. The YCs demonstrated a sense of empowerment in their actions – speaking to local businesses and organisations about the initiative, presenting to peers and local leaders, and advocating for new programs and services for youth. Overall, there appeared to be a growing sense of ownership for the initiative among the YCs and the larger community.

#### Supporting continued action

In the spring of 2014, the research team began working with youth and adult stakeholders to establish a sustainability plan for the SONAR initiative. We reached out to key contacts seeking a new, community-based facilitator for the weekly meetings. Mentorship was offered by the research team to prepare a new facilitator – this task turned out to be challenging. One of the local youth workers and a contract employee from the city office volunteered; however, their availability was limited and did not align with the YC’s availability. Leadership for the SONAR team was inconsistent for the next several months; however, in the fall of 2014, one of the YCs advocated for the project and was able to get commitment from the school-based youth worker to help facilitate project meetings and activities. In addition, other adult stakeholders have continued to provide their support when available. SONAR activities have begun to move forward again with a number of new projects underway; evidence of the community’s capacity to overcome challenges and create action to meet their needs for continued youth mental health promotion. Another challenge for sustainability was maintaining commitment from the YCs themselves, who, without research funds, would no longer be able to rely on the position as a source of income. A number of the youth left the project when financial support for involvement was no longer available; however, the group has also engaged nine new YCs. The research team offered continued support to the YCs (e.g. assistance with grant writing, support for training new YCs); however, the existing group was confident in their capacity to manage the project and to provide leadership to new members. Further, the adult facilitator who joined the group brought expertise in youth engagement and was trusted by young people in this community, which helped to maintain youth involvement. As the project moves forward in the community, decisions will have to be made regarding recruitment of new YCs and adult stakeholders, as well as future directions for the initiative. These decisions should be shaped by project evaluation data and considerations regarding feasibility.

### Step 5 – Embedding and translating

During this step, the study context is described as internalising the intervention to inform ongoing change and the research context translates evidence from the process back to the study and scientific communities [2].

#### Period of transition and efforts to promote sustainability

At the time of publishing this article, this process remains in progress. The SONAR team continues to make efforts to foster sustainability of the project following the transition from being part of a research study to being internalised as a community-developed initiative. The SONAR initiative has been featured in additional news articles in the local Lakeview newspaper and, further, the YCs were interviewed by a national radio station and have been invited to speak about their experiences throughout their community. In addition, the SONAR team (independent of the research team) has successfully achieved three grants to fund ongoing work, including $50,000 to support the development of a youth theatre program in Lakeview. The theatre program will provide important opportunities to support youth connectedness and the youth have decided to focus their first production on mental health in an effort to decrease related stigma. The research team circulated details about a province-wide youth mental health conference and assisted the new facilitator and one of the YCs in attending. This provided an opportunity for the SONAR team to build supportive relationships with other groups working to enhance mental health among young people and to identify additional evidence-based mental health resources to use in their work in the community.

The research team has made presentations highlighting aspects of the study at a number of scientific conferences and professional symposia focusing on both KT and youth mental health. Brief reports of early findings have been compiled and shared with the Lakeview community. Several scholarly publications are underway to disseminate findings to strengthen the evidence base for CBKT research and to inform future CBKT efforts. This final step of the co-KT framework should be considered an ongoing process. Internalising practices within a community setting can be slow and require ongoing efforts [11, 21]. The amount of time that this process takes will depend on a variety of characteristics within the study context, such as perceived ease and benefit of the intervention, available resources, motivation and commitment to change.

#### Advancing CBKT: What our model adds to the evidence base

**Fig. 3:**
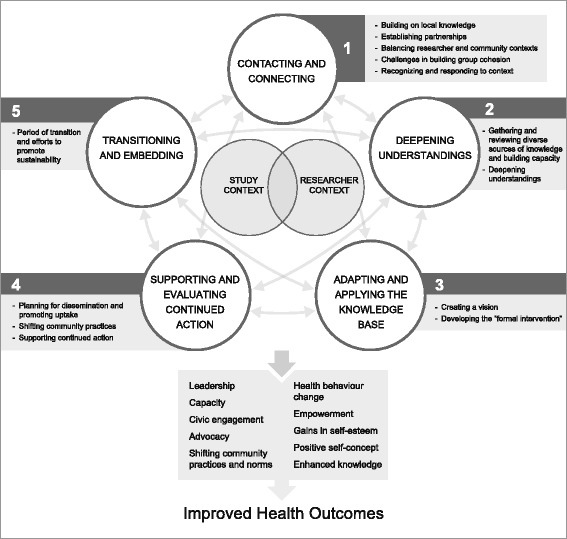
The CollaboraKTion framework is a theory driven and evidence informed framework for conducting community-based knowledge translation and builds upon existing research from the KT and participatory research fields

Our findings demonstrate that, while the co-KT framework can provide valuable guidance for researchers and practitioners engaged in CBKT, it can be advanced through adaptation based on this study. Specifically, the five steps identified by Kitson et al. [2] are more accurately represented as an iterative process involving five overarching activities: (1) contacting and connecting; (2) deepening understandings; (3) adapting and applying the knowledge base; (4) supporting and evaluating continued action; and (5) transitioning and embedding knowledge. Our analysis also supported us in identifying key process elements within each set of activities (which we have presented as sub-headings within our results section). These key elements can be viewed as important contextual considerations and outcomes related to the CBKT process and have been incorporated into the CollaboraKTion framework (Fig. 3). Our framework is also informed by central outcomes identified through the initial analysis of systematic review findings from the participatory research and KT fields.

## Discussion

CBKT is an emerging science and practice aimed at engaging populations in using knowledge production and product to build capacity for improved health outcomes. In this article, we provide a detailed overview of the activities involved in conducting a CBKT initiative and present a CBKT framework that builds upon Kitson et al.’s work [2], with the incorporation of important empirically derived key process elements and outcomes.

While the co-KT framework depicts CBKT as a stepwise linear process, our adjustments highlight the iterative or non-linear nature, wherein movement back and forth between the discrete ‘steps’ prevails to reveal processes within and across the five elements. The context within community settings is also diverse, ever shifting the nature of collaborative work and demanding team adaptability to an array of circumstances and challenges. Our team often had to revise plans and, at times, revisit activities that had already been taken. For example, we found that we repeatedly returned to Step 1 – initial contact and refining the issue (or what we have now termed, ‘contacting and connecting’), as new stakeholders were engaged or changes occurred in the study setting. In addition, as new knowledge was identified, the research team returned to processes undertaken during Step 2 – knowledge refining and testing, ensuring that this information was translated to the community to inform the study focus and resulting formal initiative. The iterative nature of our process is deeply aligned with prominent KT models used to inform evidence-based change among health practitioners (e.g. the ‘knowledge to action process’ introduced by Graham et al. [25]).

The process of CBKT is complex and the emergent nature of the work can make the activities and key processes inherently ‘messy’ for researchers who are used to working with more developed research plans. The CBKT successes are contingent on the researchers remaining flexible, making adjustments to the study ‘plan’, and coming to terms with feelings of unease that can result from the ambiguity, delays and uncertainty that can flow from working with community-based partners. In addressing these issues, our research team and YCs had frequent discussions about expectations which in turn catalysed changes to the strategy. These experiences also demonstrated that the two contexts, study and researcher, are connected (and ideally overlapping) as opposed to separate; each shaping and responsive to the other as the study progressed.

One of the key differences between KT targeting change in clinical settings and CBKT is the nature of the process and outcomes sought. While KT in clinical environments is typically aimed at creating pre-determined changes to clinicians’ practices and/or patient outcomes in an effort to better align care with an effectual intervention based on scientific evidence [1], our view of CBKT is that it should be less focused on enhancing uptake of a specific intervention and, instead, aim to collaboratively identify and create changes within the community setting that have been shown to influence health outcomes. This approach is supported by research from the sustainability literature on large system transformation in which the intervention goal is to create a culture of continuous improvement or change, acknowledging that specific practices may need to continually shift in response to changes in context [26]. Miller and Shinn [27], who write from a community psychology perspective, also provide support for this approach. In response to the issues regarding research design and the nature of valid evidence in community settings, Miller and Shinn [27] suggest developing and studying interventions while focusing on “*powerful theoretical ideas*” as opposed to attempting to create one-size-fits-all interventions. Identifying the core elements or “*active ingredients*” necessary for successful interventions is key because “*the core principles underlying an intervention, the content of the intervention, and the procedures for implementation may be transferable, but that the totality of the program is an inherently local, unique, and immovable commodity*” [27] (p. 176). With this in mind, instead of simply focusing on app utilisation, which was developed based on evidence and the experiential knowledge of the YCs communication and help-seeking strategies, the evidence-based change that was co-identified as the target for this CBKT process was enhanced community connectedness, a key factor influencing adolescent mental health [19, 21, 28].

Additionally, through the CBKT process, we were interested in promoting and achieving sustainability in community capacity, empowerment, competence in utilising diverse forms of knowledge to inform community practices, and shifting behaviour and cultural norms – all of which we expected could be achieved given results from the theoretical and empirical literature from the KT and participatory research fields (e.g. [9, 13–15, 26, 29]). It is noteworthy that although there are clear connections between participatory research and CBKT, the two approaches are distinct. While collaborative methods and community engagement are central to both participatory research and CBKT, the aim of CBKT is to utilise various sources of knowledge in order to support the development, implementation and evaluation of evidence-informed, context-relevant change to enhance the health of populations in community settings – a goal and approach that is not necessarily a requirement of participatory research.

It is also important to comment on some of the limitations of this study. Our results represent analyses based predominantly on data derived within the researcher context. While efforts were made to account for the perspectives of YCs by requesting that they keep a log of their experiences and reflections throughout the study, this was not an activity that took precedence amid their competing time demands. For example, given contextual factors within this community, such as economic instability, many young people had to take on regular employment in order to assist with family expenses. Responsibilities such as this likely contributed to fluctuating engagement of YC’s throughout the study and to reflection logs not being maintained. Similar challenges have been documented by others engaged in participatory research (e.g. [30]). Furthermore, the timeline for this study was relatively short for a community-based study [14]. Additional time in the field to engage further with the study context would have allowed greater opportunities for relationship building, a critical component to the success of CBKT. While the small size and rural nature of this community facilitated the study of CBKT processes and provided insights into the feasibility of conducting a collaborative study through a primarily virtual platform, having an adult stakeholder from the study context assisting with the weekly meetings and facilitating youth action would have likely benefited this initiative. Another limitation was the absence of health-related policymakers as community partners. While efforts were made to engage policymakers within the local health authority, our study team encountered difficulties in getting commitment from this stakeholder group. Future efforts focusing on ensuring buy-in from this and other policymaker groups are important for supporting broad changes that promote health at a population level [31]. To support this buy in, early involvement of policymakers as partners in the CBKT process should be prioritised as should training of community members in strategies needed to navigate the policy arena and effectively communicate with policymakers at various levels of government (municipal, provincial, federal). These strategies could support the ‘co-production’ of policies and services addressing the social and structural determinants of health to achieve enhanced health at a population level [32]. Despite these limitations, our findings and framework offer valuable insights and guidance for researchers planning and undertaking CBKT initiatives.

The emerging field of CBKT will require additional careful methodological considerations. While we are not calling for methodological orthodoxy, the key parameters of a valid approach need to be articulated. Through our analysis we have contributed to the methodological basis of CBKT by revising and advancing an empirically informed theory rich framework that offers practical guidance toward process application. Additional work is needed to further test the application of the CollaboraKTion framework in diverse settings and distil meaningful metrics to evaluate the ‘fit’ and rigorously account for and formally report on the outcomes garnered through CBKT. Further, while we have presented CBKT as a process which would be used by members of the research community, this approach holds relevance for health- and community wellbeing-oriented professionals versed in the use and application of research (e.g. nurses, social workers, public health professionals) and who are engaged in working with communities to enhance population health.

## Conclusions

The results of this study demonstrate that, although CBKT is a complex and often unpredictable process, it holds value for guiding the development, implementation and evaluation of evidence-informed and contextually relevant initiatives addressing the health of communities and/or populations. The CollaboraKTion framework presented in this article provides much needed direction to CBKT researchers and practitioners, but requires ongoing empirical testing across diverse settings as a means to further advancing the field.

## Abbreviations

CBKT: Community-based knowledge translation
KT: Knowledge translation
YC: Youth collaborator
